# *Reply to ‘*Emergence of *Streptococcus pneumoniae* serotype 19A (Spn19A) in the pediatric population in Bogotá, Colombia as the main cause of invasive pneumococcal disease after the introduction of PCV10’

**DOI:** 10.1080/21645515.2020.1784654

**Published:** 2020-07-09

**Authors:** Javier Nieto Guevara, Patricia Izurieta

**Affiliations:** a Medical Department, GSK, Panama City, Panama; b Medical Affairs, GSK, Wavre, Belgium

We read with interest the article titled ‘Emergence of *Streptococcus pneumoniae* serotype 19A (Spn19A) in the pediatric population in Bogotá, Colombia as the main cause of invasive pneumococcal disease after the introduction of PCV10ʹ by Germán Camacho Moreno and colleagues.^1^ This is an interesting descriptive evaluation of changes in serotype 19A prevalence in pediatric invasive pneumococcal disease (IPD) cases admitted to 10 private and state hospitals in Bogotá, Colombia, between 2008 and 2017. This period spans the introduction of the 10-valent pneumococcal non-typeable *Haemophilus influenzae* protein D conjugate vaccine (PHiD-CV, also called PCV-10) into the National Immunization Program (NIP) in January 2012.

We would like to highlight some aspects of this study, and to reflect on some issues relating to the evaluation of the impact of pneumococcal conjugate vaccines (PCV) after inclusion in NIPs.

First, the study reported by Camacho Moreno and colleagues^1^ was conducted only in a group of 10 tertiary care hospitals in the metropolitan area of Bogotá. As specified in the paper, some of these hospitals were private and others were state hospitals. This could introduce a potential for bias, since differences in medical practice and standard of care may be expected. As the study was conducted in only one city, this population may not be representative of other regions and settings, and therefore the results may not be indicative of results at a national level. Data results are presented by the number of IPD cases and percentages for cumulative periods that are not comparable; for example, 2008–2011 (4-year period), 2012–2013 (2-year period) and 2014–2017 (4 year-period). In addition, results by age groups and percentages are not consistently presented throughout the text. Although the authors claim strengthening of surveillance at the regional level, provide study population data in Bogotá and have access to data on the population aged <5 years, it is unclear why incidences by age group are not calculated. Age-specific incidence data could provide a more accurate view of the burden of overall and serotype 19A IPD.

Second, the authors calculated a mortality rate of 10.7% for serotype 19A IPD in children and young people aged <18 years (6 deaths among 56 serotype 19A IPD cases across the study period), with 83.3% of the deaths (5 of 6 deaths) occurring in children aged <5 years. It is unclear why the authors use inconsistent denominators, ages, and time periods to calculate and present the results. Furthermore, they comment that mortality rates are higher than the mortality rate reported for all IPD in a cited study published in a peer-reviewed journal.^2^ However, this 10.7% could be considered misleading. Using the same denominator as Rojas and colleagues,^2^ all IPD cases, the mortality rate due to serotype 19A would be 1.3% in children aged <18 years (6 deaths among 463 IPD cases). Using only IPD cases with known serotypes as the denominator, which is presented by age group in the paper by Camacho Moreno and colleagues,^1^ the mortality rate due to serotype 19A would be estimated at 2.1% in children aged <5 years (5 deaths among 237 IPD cases with known serotypes) and 1.9% in children aged <18 years (6 deaths among 317 IPD cases with known serotypes). The calculation method used by Camacho Moreno and colleagues^1^ is closer to the calculation of a case-fatality rate, which is not the same as the mortality definition used by Rojas and colleagues.^2^

Camacho Moreno and colleagues^1^ claim that this study provides information on the epidemiological behavior of serotype 19A in the pediatric population of Bogotá, showing a clear increase in frequency and resistance to antibiotics. However, the authors recognize as a limitation of the study that the retrospective character of the data prior to PHiD-CV introduction makes it difficult to make comparisons with the prospective analysis after the introduction. Over time, surveillance has strengthened the reporting of IPD at the regional level; however, missing serotype data remain a further limitation of the study. At best, the data in this paper provide information that serotype 19A IPD is still prevalent in children aged <5 and <18 years in Bogotá. However, the way the results are presented does not provide a clear view of the burden of disease, and the reader cannot confirm that there is a change in the serotype 19A epidemiology.

The impact of the two higher-valent PCVs (PHiD-CV and PCV-13) on overall pneumococcal disease has been established in many studies^3,4^ and the effectiveness of both vaccines against IPD is recognized by the World Health Organization (WHO)^5^ and the Pan American Health Organization (PAHO) Technical Advisory Group on Vaccines.^6^ Both WHO and PAHO recognize that there is no known evidence of a difference between the two higher-valent PCVs in their impact on overall pneumococcal disease burden.^6^

A recently conducted pharmacoeconomic analysis estimated cost savings of US$34 million per year in Colombia following the inclusion of PHiD-CV in the Colombia NIP in 2012.^7^ The same analysis indicated that switching to PCV-13 in the NIP instead of PHiD-CV would require an additional investment of US$4.4 million per year, and the estimated mean cost per serotype 19A IPD case avoided would be US$45,916 to US$107,138, depending on the vaccine efficacy against serotype 19A.^7^ Similar results were reported from a study in Brazil, which also found that switching from PHiD-CV to PCV-13 in the NIP would incur high additional costs per IPD case avoided.^8^

In conclusion, we consider that the study by Camacho Moreno and colleagues^1^ should be interpreted with the following considerations: (1) the study population may not be generalizable to the whole population of Colombia, (2) the way of the data are presented does not allow us to confirm changes in 19A IPD epidemiology in Bogotá, and (3) the estimated mortality rate for serotype 19A IPD could be considered misleading. Perhaps the study could be evaluated in the context of other studies, which indicate that switching to PCV-13 in NIPs in Latin America would incur a high incremental cost per serotype 19A IPD case avoided, and the challenges potentially presented by that.
